# Supplementary figures and images for: A genome-wide CRISPR/Cas9 knockout screen identifies TMEM239 as an important host factor in facilitating African swine fever virus entry into early endosomes
Source: PLoS Pathog. 2024 Jul 18;20(7):e1012256. doi: 10.1371/journal.ppat.1012256 (PMC11288436; doi:10.1371/journal.ppat.1012256)

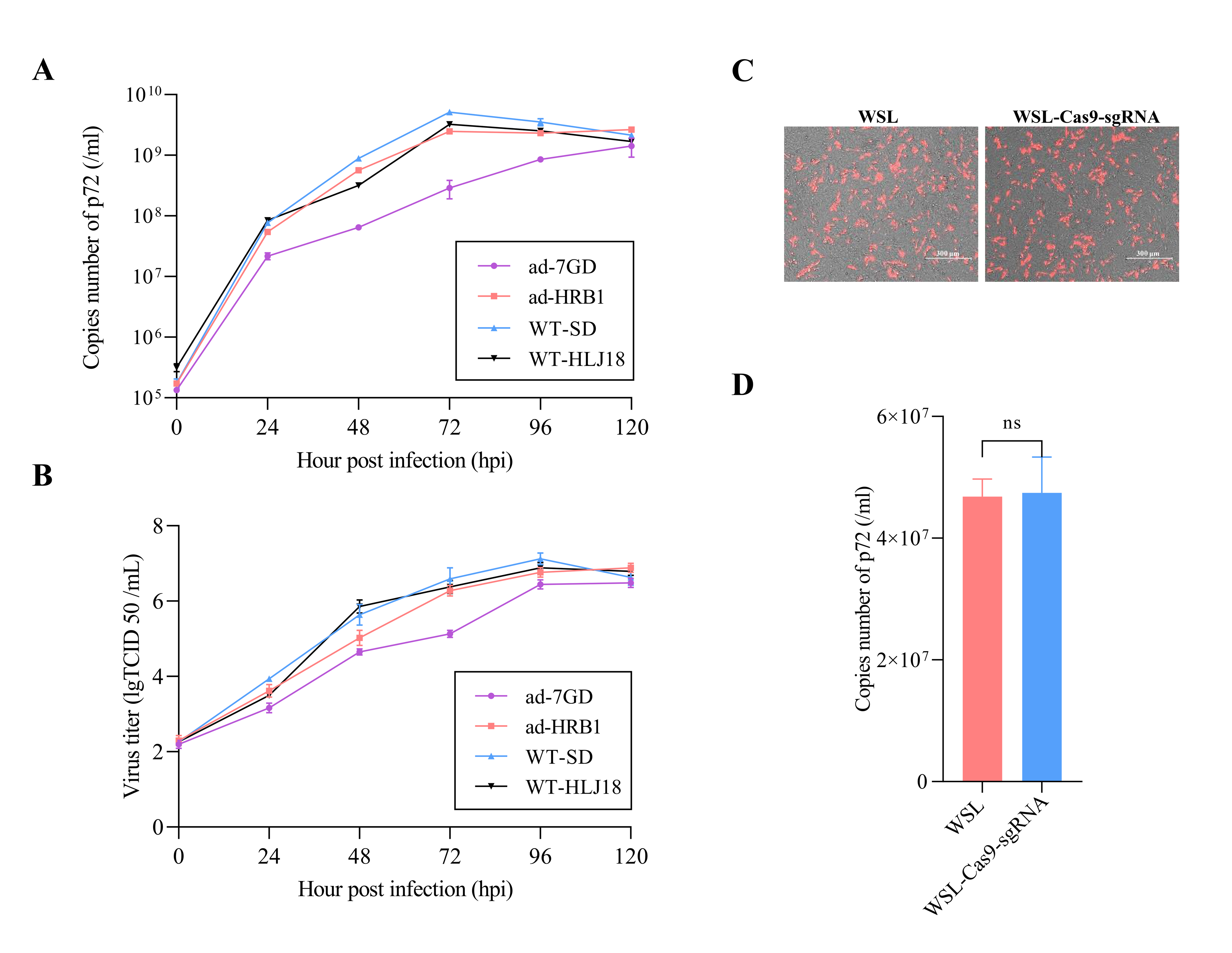

Supplement: S1 Fig — (A and B) Multistep growth curves of WSL-adapted strains (ad-7GD and ad-HRB1) and wild-type strains (WT-SD and WT-HLJ18) in PAMs. PAMs were infected with ad-HRB1, WT-SD, and WT-HLJ18 at a Multiplicity of Infection (MOI) of 0.1, while ad-7GD was infected at an MOI of 1. Samples were collected daily until cell destruction by virus infection. Viral replication was characterized by quantifying viral genome copies (p72) and viral titers. (C) Replication of ad-7GD in WSL and WSL-Cas9-sgRNA cells. Fluorescence micrographs depicting the replication of ad-7GD in WSL cells and WSL-Cas9-sgRNA cells following incubation with the virus (MOI = 1) for 48 h. (D) Quantification of viral genome copies (p72) in the cell culture supernatants of WSL cells and WSL-Cas9-sgRNA cells. ns: not significant. (TIF) [file ppat.1012256.s001.tif]

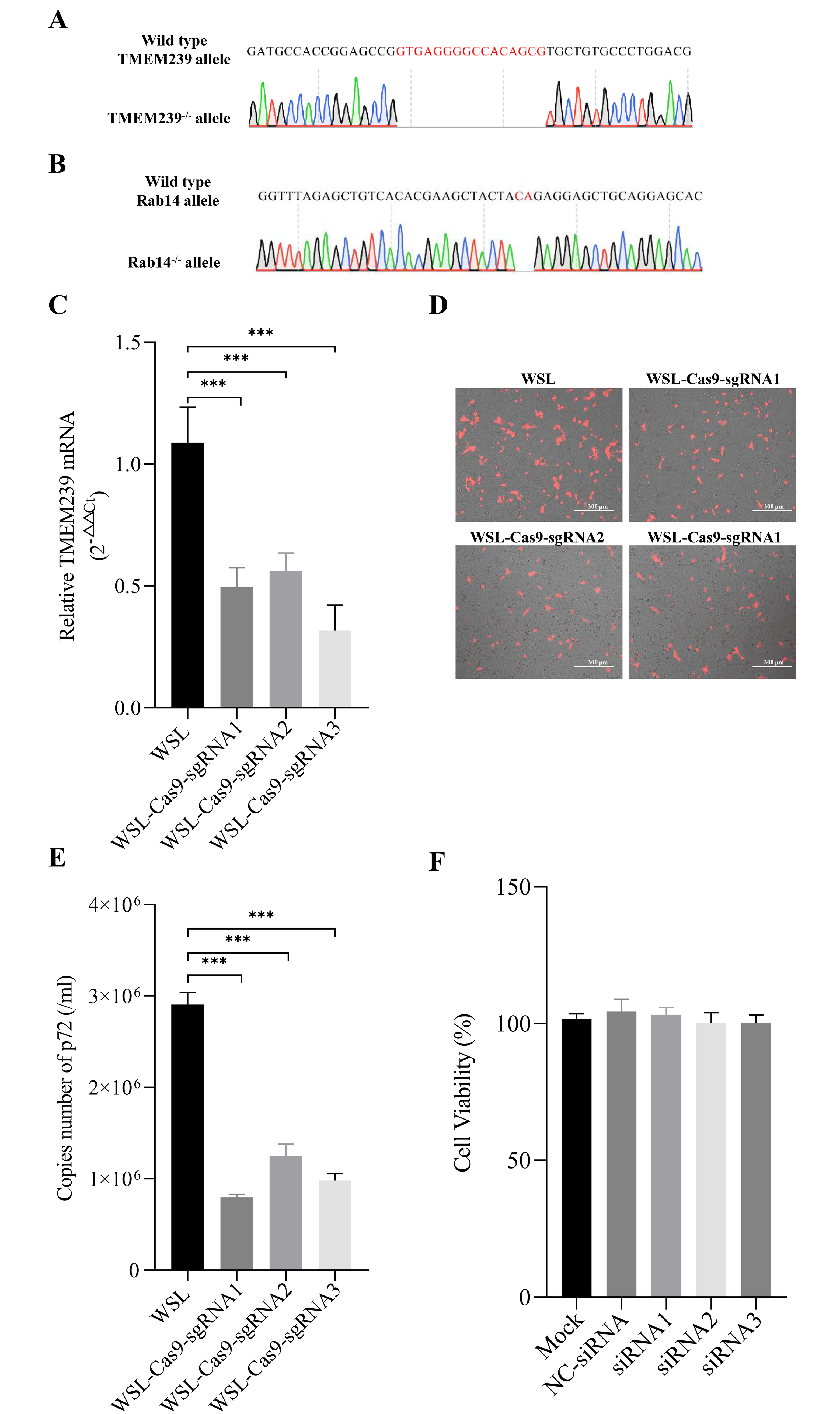

Supplement: S2 Fig — (A) Identification of TMEM239 gene monoclonal knockout cell lines through Sanger sequencing. (B) Identification of Rab14 gene monoclonal knockout cell lines through Sanger sequencing. (C) RT-qPCR analysis of TMEM239 transcription in polyclonal knockout cell lines. ***p < 0.001. (D) Replication of ad-7GD in TMEM239 polyclonal knockout cell lines. Fluorescence micrographs illustrating the replication of ad-7GD in WSL cells and various TMEM239 polyclonal knockout cell lines generated through the transfection of different sgRNAs. All cells were incubated with virus (MOI = 1), and fluorescence micrographs were captured at 48 hpi. (E) Quantification of viral genome copies (p72) in the cell culture supernatants of WSL cells and diverse TMEM239 polyclonal knockout cell lines. ns: not significant. ***p < 0.001. (F) Assessment of cytotoxic effects of siRNAs on PAMs. PAMs were individually transfected with all siRNAs and incubated for 24 h. Then, the influence of these siRNAs on cell viability was assessed using the CCK8 assay. (TIF) [file ppat.1012256.s002.tif]

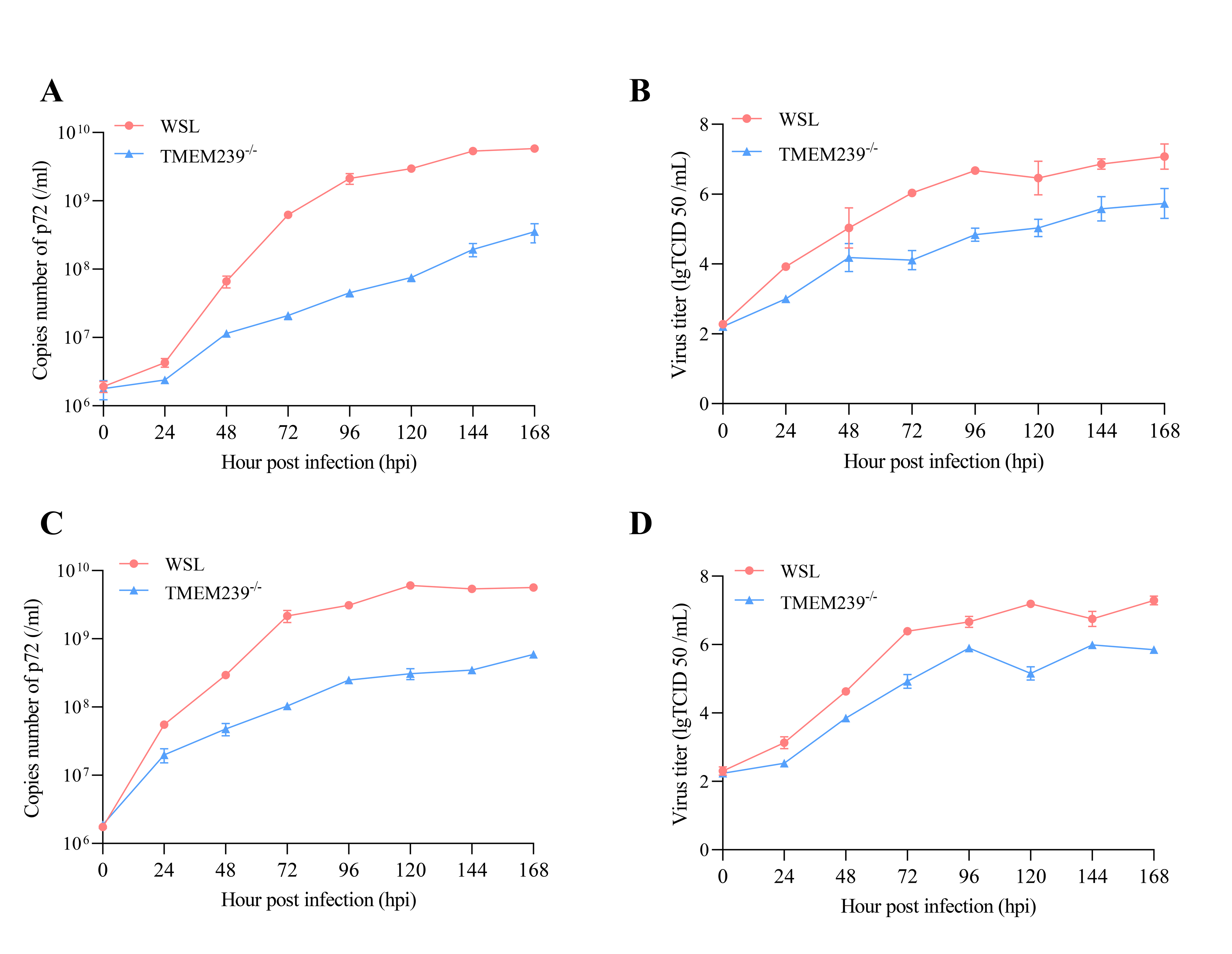

Supplement: S3 Fig — (A and B) Multistep growth curves of ad-HRB1 in WSL cells and TMEM239-/- cells. Cells were infected with ASFV Genotype II ad-HRB1 strain (MOI = 1), and samples were collected daily until cell destruction by the virus. Viral replication was characterized by quantifying viral genome copies (p72) and viral titers. (C and D) Multistep growth curves of WT-SD in WSL cells and TMEM239-/- cells. Cells were infected with ASFV Genotype I WT-SD strain (MOI = 1), and samples were collected daily until cell destruction by the virus. Viral replication was characterized by quantifying viral genome copies (p72) and viral titers. (TIF) [file ppat.1012256.s003.tif]

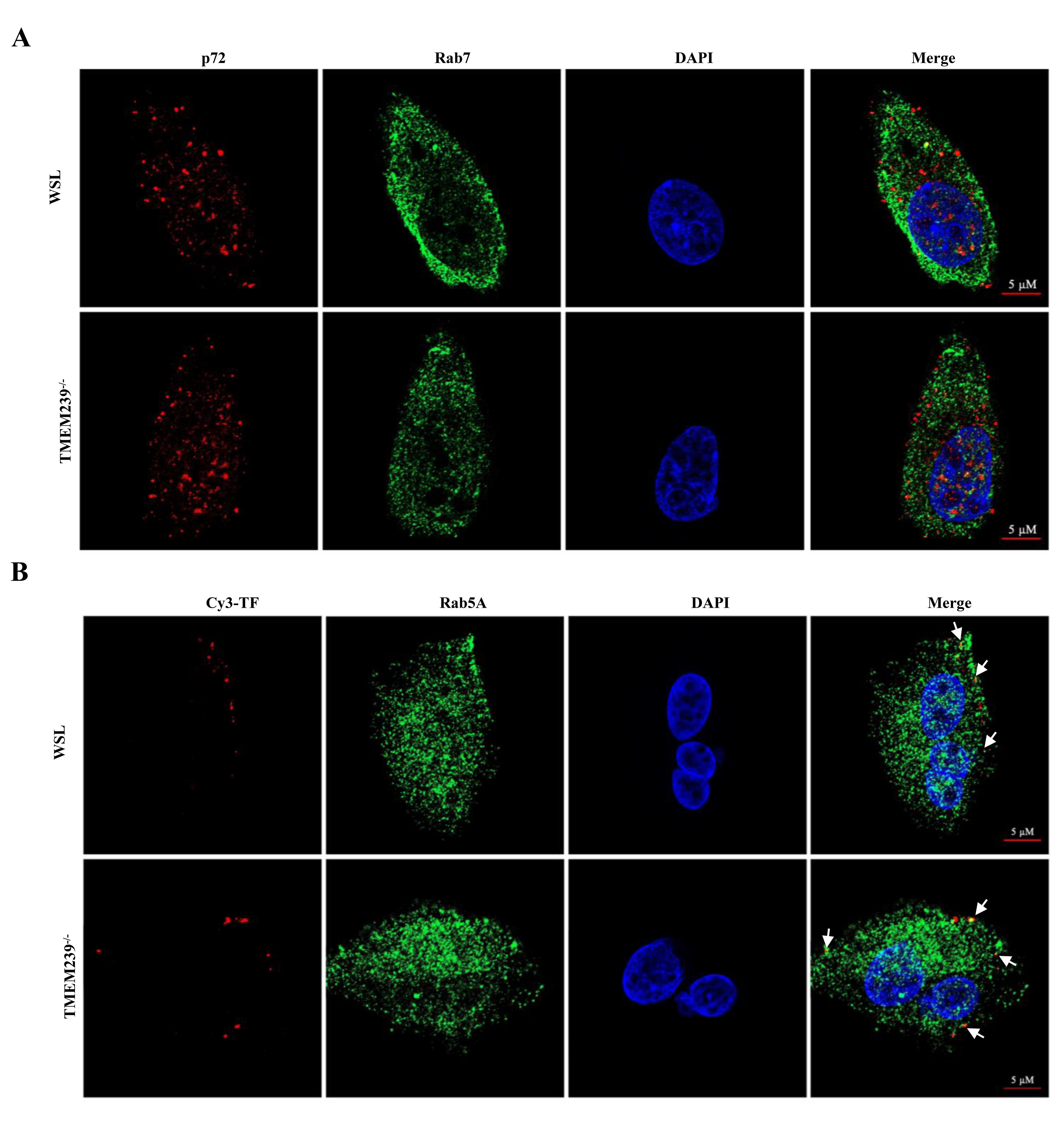

Supplement: S4 Fig — (A) Colocalization analysis of viral p72 and Rab7 was performed at 45 minutes after viral infection. WSL cells and TMEM239-/- cells were incubated with ad-HRB1 isolates (MOI = 20) for 2 h at 4°C and then washed with ice-cold PBS to remove unbound virus particles. Then, ice-cold medium was added, and the cells were incubated at 37°C for 45 minutes before being fixed in methanol. Immunofluorescence analysis was performed against p72 (red) and Rab7 (green). Nuclear DNA was visualized by DAPI staining (blue). (B) Transferrin uptake assay in WSL cells. After serum starvation for 30 minutes, WSL cells were incubated with 50 μg/mL Cy3-labelled transferrin in DMEM for 20 minutes at 4°C for binding. Subsequently, cells were washed with ice-cold PBS to remove unbound protein, shifted to 37°C for 20 minutes to facilitate transferrin internalization, and subjected to trypsin treatment to eliminate remaining surface-bound protein before fixation in methanol. Immunofluorescence analysis was performed against transferrin (red) and Rab5A (green). Nuclear DNA was visualized by DAPI staining (blue). The arrows indicate transferrin transferred to early endosomes. (TIF) [file ppat.1012256.s004.tif]

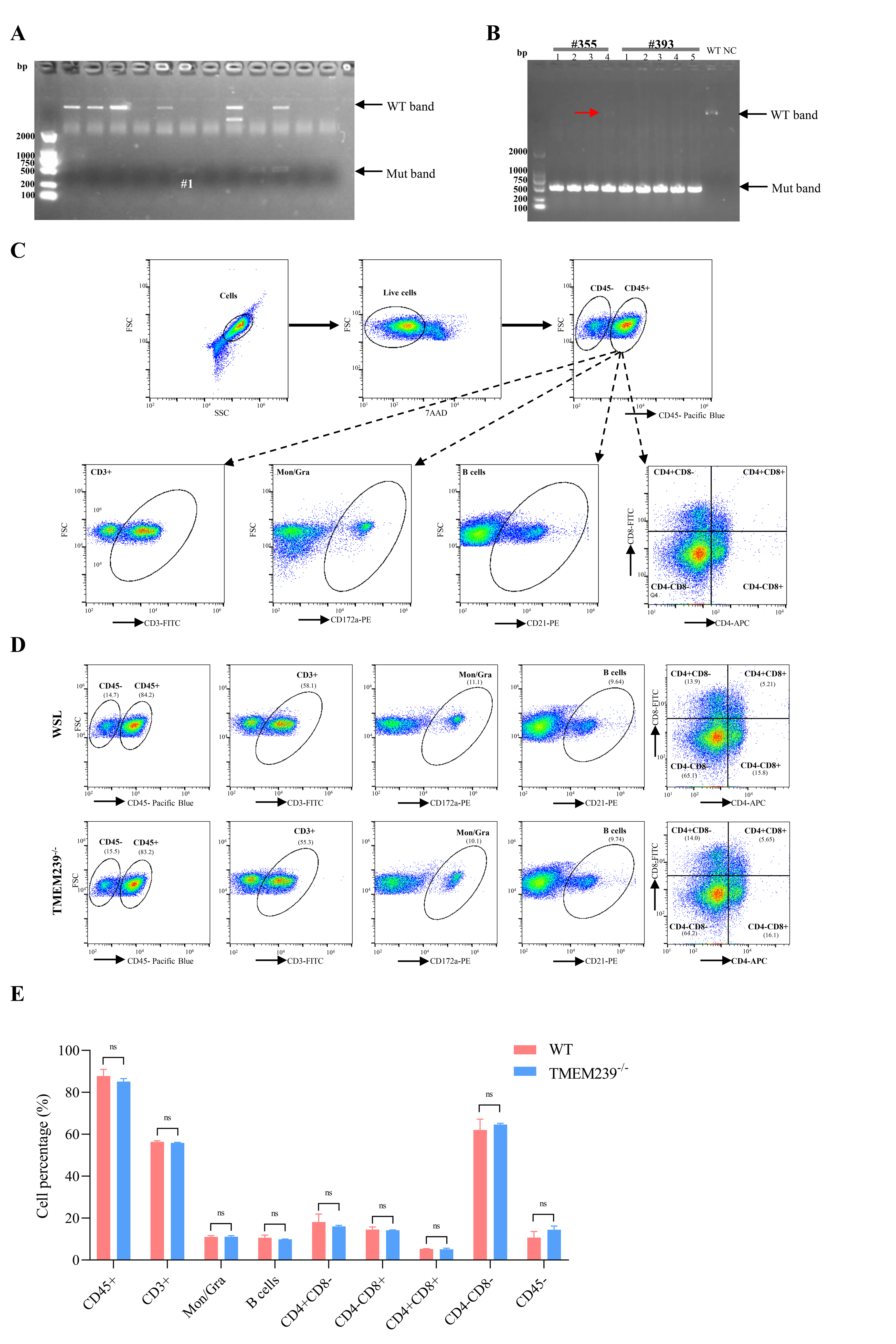

Supplement: S5 Fig — (A) Identification of TMEM239 gene monoclonal knockout PEF cell clones through PCR. Primer pair F3/R4 was used to identify PEF cell clones generated by the CRISPR/Cas9 gene editing system with complete deletion of the coding region of the TMEM239 gene. Specifically, primer pair F3/R4 amplifies a 7678-bp band in wild-type PEF cells, whereas in PEF cells, in which the coding region of the TMEM239 gene is entirely deleted, a ~513-bp band is amplified. Clone #1 represents a PEF cell clone with complete deletion of the coding region of the TMEM239 gene. (B) Genotype identification of SCNT-producing piglets. DNA was extracted from piglet ear margins for PCR identification. Primer pair F3/R4 was used to identify piglet genotypes. Primer pair F3/R4 amplified a 7678-bp band in wild-type piglets and a ~513-bp band in TMEM239 homozygous knockout piglets. The red arrow indicates the wild-type bands from piglet #355–4. WT, Wild-type piglet, NC, Negative control. (C) Gating strategy of the proliferation assay. 7-aminoactinomycin D (7-AAD) was utilized to identify live cells, while anti-pig CD45 was employed to isolate lymphocytes. Subsequently, based on CD45+, cells were analyzed for the presence of CD3+, Monocyte/Granulocyte (Mon/Gra), B cells, CD4+CD8-, CD4-CD8+, and CD4+CD8+ populations. (D and E) Results of flow cytometry analysis on PBMCs from TMEM239-/- pigs and wild-type pigs. PBMCs from TMEM239-/- pigs and wild-type pigs were surface-labeled with anti-porcine CD45 (Pacific Blue), anti-porcine CD3ε-FITC, anti-porcine 172a-PE, anti-porcine CD21-PE, anti-porcine CD4-APC, anti-porcine CD8-FITC, and anti-porcine CD21-PE. (TIF) [file ppat.1012256.s005.tif]

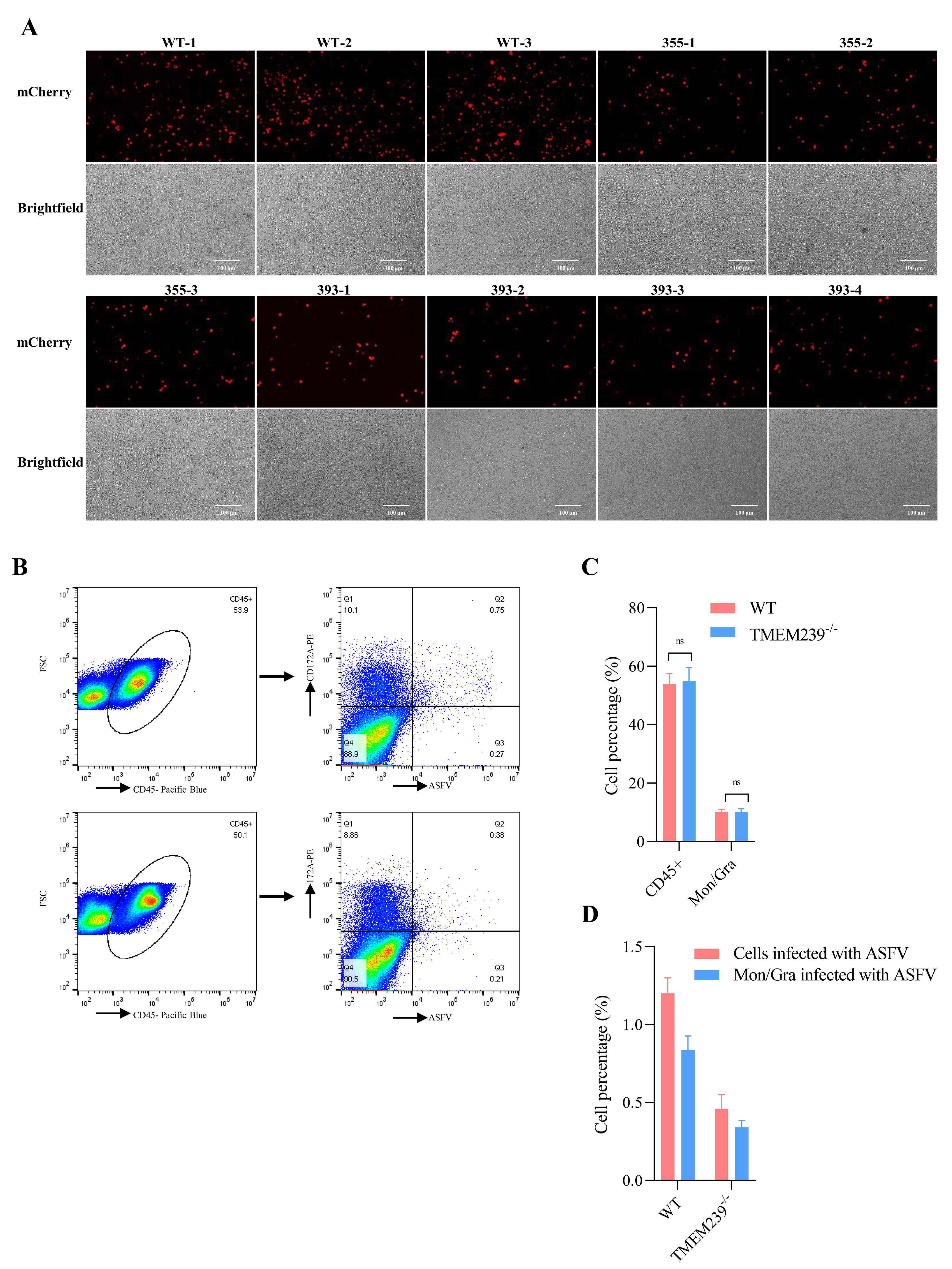

Supplement: S6 Fig — (A) Fluorescence micrographs illustrating ad-7GD replication. WT PBMCs and TMEM239-/- PBMCs were individually infected with the ad-7GD strain at an MOI of 1. Fluorescence micrographs were collected at 72 hpi. WT-1, WT-2, and WT-3 were the numbers of the wild-type piglets selected for the current experiment. Piglets numbers 355-(1–4) and 393-(1–5) correspond to piglets born to surrogate sows #355 and #393, respectively. (B-D) Flow cytometric analysis of PBMCs infected with ASFV. WT PBMCs and TMEM239-/- PBMCs were individually infected with the ad-7GD strain at an MOI of 1 for 16 h. These PBMCs were surface-labeled with anti-porcine CD45 (Pacific Blue) and anti-porcine 172a-PE. Meanwhile, a gate was set to detect ASFV infected cells. (TIF) [file ppat.1012256.s006.tif]
