# Supplementary material for: A genome-wide CRISPR/Cas9 knockout screen identifies TMEM239 as an important host factor in facilitating African swine fever virus entry into early endosomes
Source: PLoS Pathog. 2024 Jul 18;20(7):e1012256. doi: 10.1371/journal.ppat.1012256 (PMC11288436; doi:10.1371/journal.ppat.1012256)
Supplement: S1 Table — (DOCX) [file ppat.1012256.s007.docx]

**S1 Table. Information of ASFV strains used in this study.**

| **Virus** | **Genotype** | **Accession number** | **Parental strain** | **Adaptation in WSL cells** | **No. of passages in WSL cells** | **CPE in WSL cells** | **Gene deletion** |
| --- | --- | --- | --- | --- | --- | --- | --- |
| HLJ/18  (WT-HLJ18) | II | MK333180 | Natural isolate [7] | NO | / | NO | / |
| SD/DY-I/21  (WT-SD) | I | MZ945537 | Natural isolate [31] | NO | / | YES | MGF110-11L/12L, MGF360-6L/10L/11L/12L/13L/14L, MGF505-1R/2R, MGF360-9L, MGF505-3R, EP153R, EP402R and MGF100-2L. |
| ad-HRB1 | II | PP355086 | HLJ/HRB1/20 [32] | YES | 30 | YES | MGF360-1L/2L/3L, MGF110-1L/2L/3L/4L/5L/6L/7L, I7L and I9R. |
| ad-7GD | II | PP355087 | HLJ/18-7GD [34] | YES | 25 | YES | MGF505-1R/2R/3R, MGF360-12L/13L/14L, EP402R, I7L, I8L, I9R, I10L and L11L. |
