# Supplementary material for: A genome-wide CRISPR/Cas9 knockout screen identifies TMEM239 as an important host factor in facilitating African swine fever virus entry into early endosomes
Source: PLoS Pathog. 2024 Jul 18;20(7):e1012256. doi: 10.1371/journal.ppat.1012256 (PMC11288436; doi:10.1371/journal.ppat.1012256)
Supplement: S2 Table — (DOCX) [file ppat.1012256.s008.docx]

**S2 Table. The sequences of sgRNAs, siRNAs and primers used in this study.**

| **siRNA** | **Sequence (5'-3')** |
| --- | --- |
| TMEM239-siRNA1 | GGGUCUGGAAGGGAUCCUCTT |
| TMEM239-siRNA2 | GCUCUGCCAUGCACUCUUCTT |
| TMEM239-siRNA3 | GCUCUUCAUGGCCUCCUUCTT |
| NC-siRNA | UUCUCCGAACGUGUCACGUTT |
| **sgRNA** | **Sequence (5'-3')** |
| Rosa26-locus-sgRNA | GTGAGAGTTATCTGACCGTAAGG |
| TMEM239-sgRNA | AGCACGCTGTGGCCCCTCACCGG |
| Rab14-sgRNA | GTCACACGAAGCTACTACAGAGG |
| TMEM239-sgRNA1 | GCCCAGTGGTGGATCACATCGGG |
| TMEM239-sgRNA2 | GTGCCACATGCCCCCGAGCTGGG |
| TMEM239-sgRNA3 | ATGCCACCGGAGCCGGTGAGGGG |
| PEF-KO-sgRNA1 | AGACTAGCCACCCCAACCTACGG |
| PEF-KO-sgRNA2 | GTAGCAGAGTCACAAACCTAGGG |
| **Primers** | **Sequence (5'-3')** |
| TMEM239-Flag-F | GGCAAAGAATTGCCACCGTGATGCAGCAGCTGCGAG |
| TMEM239-Flag-R | GTCGTCGTCCTTGTAGTCTTGGTCCAAGTCCTGAGCAAGGC |
| TMEM239-Myc-F | GGCAAAGAATTGCCACCGTGATGCAGCAGCTGCGAG |
| TMEM239-Myc-R | TCAGAAATGAGCTTTTGCTCTTGGTCCAAGTCCTGAGCAAGG |
| Rab5A-F | GGCAAAGAATTCGCCACCATGGCTAATCGAGGAGCAACAAG |
| Rab5A-R | AGAAATGAGCTTTTGCTCGCCGGATCCGTTACTACAACACTGAC |
| B646L-F | GGCAAAGAATTCGCCACCATGGCATCAGGAGGAGCTTTTTG |
| B646L-R | ATCTGGTACGTCGTATGGGTAGCCGGATCCGGTACTGTAA |
| O61R | GGCAAAGAATTCGCCACCATGGCACTTGATGGTTCAAGTGG |
| O61R | GTCGTATGGGTAGCCGGATCCACTTGTTTTTAGGGAACAGCT |
| E248R | GGCAAAGAATTCGCCACCATGGGAGGCTCTACAAGCAAAAAT |
| E248R | GTACGTCGTATGGGTAGCCGGATCCCGAAACGGCAGCATTTTTTA |
| B438L | GGCAAAGAATTCGCCACCATGTATCATGATTATGCTTCAAAGC |
| B438L | CTGGTACGTCGTATGGGTAGCCGGATCCCAATGATGGAGATAT |
| E183L | GGCAAAGAATTCGCCACCATGGATTCTGAATTTTTTCA |
| E183L | TGGTACGTCGTATGGGTAGCCGGATCCCAAGGAGTTTTCTA |
| D117L | GGCAAAGAATTCGCCACCATGGACACTGAAACGTCTCCACTG |
| D117L | TCTGGTACGTCGTATGGGTAGCCGGATCCTGAATGCGCAAGT |
| E199L | GGCAAAGAATTCGCCACCATGTCTTGCATGCCAGTTTCC |
| E199L | AATCTGGTACGTCGTATGGGTAGCCGGATCCAAAATTGTTTAG |
| E120R | GGCAAAGAATTCGCCACCATGGCAGATTTTAATTCTCCAAT |
| E120R | TCTGGTACGTCGTATGGGTAGCCGGATCCCTTCGATTTATGCG |
