# Supplementary material for: A genome-wide CRISPR/Cas9 knockout screen identifies TMEM239 as an important host factor in facilitating African swine fever virus entry into early endosomes
Source: PLoS Pathog. 2024 Jul 18;20(7):e1012256. doi: 10.1371/journal.ppat.1012256 (PMC11288436; doi:10.1371/journal.ppat.1012256)
Supplement: S6 Table — (DOCX) [file ppat.1012256.s012.docx]

**S6 Table. Identification of TMEM239 binding proteins in the ASFV uninfected group by use of mass spectrometry.**

| **Accession** | **Gene name** | **MW [kDa]** | **Coverage [%]** | **No. of PSMs^*^** | **No. of Peptides** | **No. of Unique Peptides** | **Score** |
| --- | --- | --- | --- | --- | --- | --- | --- |
| A0A8D0ZZA4 | CAP1 | 51.2 | 30 | 12 | 11 | 11 | 35.33 |
| A0A8D1FNA5 | SCYL2 | 103.5 | 17 | 11 | 11 | 11 | 31.67 |
| A0A287AGU2 | ATP5F1A | 59.4 | 18 | 11 | 9 | 8 | 28.70 |
| A0A8D1G3M0 | KRT3 | 64.4 | 8 | 11 | 8 | 2 | 28.61 |
| A0A8D1HZ32 | DOCK4 | 224.4 | 4 | 6 | 6 | 6 | 15.59 |
| A0A4X1VME2 | CAPRIN2 | 113.7 | 8 | 6 | 6 | 6 | 14.99 |
| A0A8D0YQW8 | PKM | 126.2 | 6 | 6 | 6 | 6 | 14.10 |
| A0A8D1S1I4 | TUBA1A | 51.9 | 15 | 5 | 5 | 5 | 13.95 |
| A0A8D0WX08 | EPRS1 | 170.8 | 3 | 5 | 4 | 4 | 12.25 |
| A0A286ZZM8 | DHRS7B | 35.0 | 18 | 5 | 5 | 5 | 12.15 |
| A0A4X1U2X6 | RACK1 | 30.5 | 21 | 3 | 3 | 3 | 11.05 |
| A0A5G2R4I1 | PIP5K1C | 74.1 | 10 | 4 | 4 | 4 | 10.56 |
| A0A8D1H6J3 | THBS1 | 136.3 | 2 | 4 | 3 | 3 | 8.30 |
| A0A287ASI0 | KRT7 | 50.8 | 6 | 3 | 3 | 1 | 7.90 |
| Q9MZ15 | VDAC2 | 31.6 | 14 | 3 | 3 | 3 | 7.65 |
| A1XQU9 | RPS20 | 13.3 | 19 | 3 | 2 | 2 | 7.05 |
| A0A4X1TBF9 | SLC25A6 | 32.8 | 11 | 3 | 3 | 1 | 7.01 |
| P00355 | GAPDH | 35.8 | 9 | 3 | 3 | 3 | 6.90 |
| A0A8D1NDK0 | ANXA2 | 38.6 | 8 | 2 | 2 | 2 | 5.95 |
| A0A8D1P6P9 | DDOST | 53.5 | 5 | 2 | 2 | 2 | 5.66 |
| A0A287B037 | PHB2 | 32.3 | 7 | 2 | 2 | 2 | 4.99 |
| I3LAT6 | IARS1 | 144.5 | 2 | 2 | 2 | 2 | 4.87 |
| K7GNY4 | RPL8 | 24.6 | 5 | 2 | 1 | 1 | 4.76 |
| Q06AU6 | RAB5A | 23.5 | 11 | 2 | 2 | 2 | 4.50 |
| A0A8D1RBE5 | FAAH | 62.8 | 4 | 2 | 2 | 2 | 4.37 |
| Q52NJ6 | RAB14 | 23.9 | 12 | 2 | 2 | 2 | 4.31 |
| A0A287AE47 | STOM | 29.2 | 8 | 2 | 2 | 2 | 4.16 |
| A0A4X1W169 | STOML2 | 38.6 | 4 | 2 | 1 | 1 | 4.12 |
| A0A8D0QIE5 | LMO7 | 144.3 | 1 | 2 | 2 | 2 | 4.05 |
| I3LEC2 | PCBP1 | 37.5 | 7 | 2 | 2 | 2 | 3.85 |
| A0A8D0T9F3 | RPS4X | 27.6 | 5 | 1 | 1 | 1 | 3.29 |
| F2Z5L5 | H2AC20 | 14.0 | 15 | 1 | 1 | 1 | 3.27 |
| A0A8D1BGE7 | LTBP1 | 181.0 | 1 | 1 | 1 | 1 | 3.27 |
| A0A4X1U700 | RPL12 | 17.8 | 10 | 1 | 1 | 1 | 2.87 |
| A0A5G2R4W7 | SUCLG1 | 35.5 | 4 | 1 | 1 | 1 | 2.65 |
| Q29375 | RPL7A | 14.6 | 10 | 1 | 1 | 1 | 2.61 |
| A0A8D1LJ43 | PSMD2 | 98.7 | 2 | 1 | 1 | 1 | 2.54 |
| A0A8D0U9X5 | RAC2 | 24.3 | 6 | 1 | 1 | 1 | 2.42 |
| A0A286ZZ84 | ABCB5 | 131.1 | 1 | 1 | 1 | 1 | 2.41 |
| A0A8D1H5C0 | RBP1 | 15.2 | 10 | 1 | 1 | 1 | 2.40 |
| P33198 | IDH2 | 47.5 | 3 | 1 | 1 | 1 | 2.37 |
| F1S5K0 | PPM1B | 52.0 | 4 | 1 | 1 | 1 | 2.36 |
| Q9MYT8 | ATP5ME | 8.2 | 17 | 1 | 1 | 1 | 2.33 |
| A0A8D0LDX8 | UGDH | 51.3 | 2 | 1 | 1 | 1 | 2.28 |
| A0A8D1AEG5 | FARP1 | 113.1 | 1 | 1 | 1 | 1 | 2.25 |
| A0A287A4W0 | FLNA | 302.6 | 0 | 1 | 1 | 1 | 2.25 |
| A0A5G2R912 | SPART | 72.7 | 2 | 1 | 1 | 1 | 2.24 |
| A0A8D0PQB4 | TM9SF4 | 74.4 | 1 | 1 | 1 | 1 | 2.24 |
| F1SV90 | EIF3I | 30.9 | 4 | 1 | 1 | 1 | 2.23 |
| A0A8D1KSN7 | MED23 | 146.2 | 1 | 1 | 1 | 1 | 2.19 |
| B0FWK5 | RPL5 | 34.4 | 5 | 1 | 1 | 1 | 2.18 |
| A0A8D0N6M5 | ACAP2 | 85.0 | 1 | 1 | 1 | 1 | 2.18 |
| A0A4X1U6G5 | JUP | 82.0 | 1 | 1 | 1 | 1 | 2.13 |
| A0A8D1C7A6 | SAFB2 | 108.0 | 1 | 1 | 1 | 1 | 2.11 |
| A0A4X1W5F1 | MARS1 | 107.6 | 1 | 1 | 1 | 1 | 2.04 |
| A0A8D0LP66 | OLA1 | 42.4 | 2 | 1 | 1 | 1 | 2.03 |
| A0A8D1QKL0 | SMARCC1 | 124.4 | 1 | 1 | 1 | 1 | 2.03 |
| A0A287BQ02 | PABPC4 | 72.3 | 2 | 1 | 1 | 1 | 2.02 |
| A0A4X1UF06 | PSMD3 | 60.9 | 2 | 1 | 1 | 1 | 2.01 |
| A0A287BL05 | HNRNPH1 | 51.9 | 2 | 1 | 1 | 1 | 1.94 |
| A0A8D1GAC9 | ABLIM1 | 83.5 | 2 | 1 | 1 | 1 | 1.91 |
| A0A8D1N486 | SLC34A1 | 69.3 | 2 | 1 | 1 | 1 | 0.00 |
| A0A8D1A7L7 | C3 | 185.2 | 0 | 1 | 1 | 1 | 0.00 |

^*^PSMs: peptide-spectrum matches
