# Supplementary material for: A genome-wide CRISPR/Cas9 knockout screen identifies TMEM239 as an important host factor in facilitating African swine fever virus entry into early endosomes
Source: PLoS Pathog. 2024 Jul 18;20(7):e1012256. doi: 10.1371/journal.ppat.1012256 (PMC11288436; doi:10.1371/journal.ppat.1012256)
Supplement: S7 Table — (DOCX) [file ppat.1012256.s013.docx]

**S7 Table. Identification of TMEM239 binding proteins in ASFV infected group by use of mass spectrometry.**

| **Accession** | **Gene name** | **MW [kDa]** | **Coverage [%]** | **No. of PSMs** | **No. of Peptides** | **No. of Unique Peptides** | **Score** |
| --- | --- | --- | --- | --- | --- | --- | --- |
| P00355 | GAPDH | 35.8 | 16 | 4 | 4 | 2 | 11.01 |
| F1SGG6 | [KRT5](https://www.ncbi.nlm.nih.gov/gene/100511564) | 63.2 | 6 | 4 | 4 | 3 | 9.50 |
| A0A8D1XIB0 | HNRNPK | 50.9 | 12 | 4 | 4 | 4 | 9.35 |
| A0A8D1UFP2 | ENSSSCG00060002145 | 18.2 | 20 | 3 | 2 | 2 | 8.24 |
| A0A4X1T1G4 | BCAP31 | 36.6 | 11 | 3 | 3 | 3 | 8.22 |
| A0A2K6E2S6 | KRT14 | 46.5 | 7 | 3 | 3 | 2 | 7.26 |
| A0A4X1SRH0 | [EEF1A2](https://www.ensembl.org/sus_scrofa_pietrain/Transcript/Summary?t=ENSSSCT00055018928&db=core) | 50.4 | 5 | 2 | 2 | 2 | 4.94 |
| P01965 | HBA | 15.0 | 17 | 2 | 2 | 2 | 4.90 |
| Q863T6 | SLC22A1 | 55.9 | 6 | 2 | 2 | 2 | 4.73 |
| F1S5K0 | PPM1B | 52.0 | 3 | 1 | 1 | 1 | 2.85 |
| A0A8D1F028 | ARMCX2 | 66.9 | 7 | 1 | 1 | 1 | 2.75 |
| A0A4X1TSV0 | HADHA | 83.1 | 3 | 1 | 1 | 1 | 2.74 |
| Q6QN13 | DIO1 | 28.9 | 6 | 1 | 1 | 1 | 2.58 |
| A0A8D1RPM8 | TMEM239 | 17.0 | 9 | 1 | 1 | 1 | 2.57 |
| A0A4X1UCS3 | FSCN2 | 69.0 | 2 | 1 | 1 | 1 | 2.34 |
| A0A4X1VJZ1 | RP2 | 39.5 | 2 | 1 | 1 | 1 | 2.15 |
| A0A287BCW5 | RPS6 | 28.6 | 5 | 1 | 1 | 1 | 2.13 |
| A0A287BSL9 | G6PD | 59.2 | 3 | 1 | 1 | 1 | 2.11 |
| A0A287BIE4 | NEDD4 | 149.1 | 2 | 1 | 1 | 1 | 2.11 |
| Q06AU6 | RAB5A | 23.5 | 2 | 1 | 1 | 1 | 2.07 |
| A0A4X1TAR0 | PTP4A1 | 17.1 | 5 | 1 | 1 | 1 | 2.05 |
| A0A8D1QKL0 | SMARCC1 | 124.4 | 1 | 1 | 1 | 1 | 2.00 |
| F1SCT9 | RDH14 | 36.8 | 2 | 1 | 1 | 1 | 1.99 |
| A0A8D0LP66 | OLA1 | 42.4 | 2 | 1 | 1 | 1 | 1.98 |
| A0A8D0PQB4 | TM9SF4 | 74.4 | 1 | 1 | 1 | 1 | 1.98 |
| A0A8D1AC24 | BCL11A | 91.2 | 1 | 1 | 1 | 1 | 1.97 |
| A0A5G2R5V7 | USO1 | 103.8 | 1 | 1 | 1 | 1 | 1.97 |
| A0A8W4F825 | MAPK1 | 136.2 | 1 | 1 | 1 | 1 | 1.95 |
| A0A287B7V0 | PIKFYVE | 50.3 | 2 | 1 | 1 | 1 | 1.94 |
| F2Z5L5 | H2AC20 | 14.0 | 5 | 1 | 1 | 1 | 1.93 |
| A0A4X1W5F1 | MARS1 | 107.6 | 1 | 1 | 1 | 1 | 1.93 |
| A0A8D1EAK8 | CCDC138 | 66.6 | 1 | 1 | 1 | 1 | 1.92 |
| A0A8D1VUA0 | HADHB | 32.9 | 2 | 1 | 1 | 1 | 1.90 |
| I3LRU2 | ELOA | 81.2 | 1 | 1 | 1 | 1 | 1.90 |
| Q8WNW5 | CDH5 | 88.2 | 1 | 1 | 1 | 1 | 0.00 |

^*^PSMs: peptide-spectrum matches
