# Supplementary material for: A genome-wide CRISPR/Cas9 knockout screen identifies TMEM239 as an important host factor in facilitating African swine fever virus entry into early endosomes
Source: PLoS Pathog. 2024 Jul 18;20(7):e1012256. doi: 10.1371/journal.ppat.1012256 (PMC11288436; doi:10.1371/journal.ppat.1012256)
Supplement: Attachment — Submitted filename: Response to Reviewers.docx [file ppat.1012256.s014.docx]

**Response letter to Reviewers’ comments (PPATHOGENS-D-23-02109)**

**Point-by-point responses to the comments of the Editor and reviewers and a list of changes are:**

(The comments of the Editor and Reviewers are in italics and blue color, which are followed by our responses. All main revisions are highlighted using yellow color in the revised manuscript.)

**Response to Reviewer 1**

**Major concerns:**

*1.* *Use of three WSL adapted viruses that don’t appear to be characterized. What changes if any occurred during the adaptation to WSL cells and do these viruses replicate in primary macrophages?*

Thanks for the reviewer’s suggestion. As suggested, we performed deep sequencing and genomic analysis for the two cell-adapted viruses (ad-HRB1 and ad-7GD) as described in lines 133 - 143. Acturally wild type SD/DY-I/21 (WT-SD) isolated from the field [1] could replicate efficiently in WSL cells without any adaptation, and we have described this in lines 126 - 136. And these three strains (WT-SD, ad-HRB1, and ad-7GD) replicate well in primary porcine macrophages (Fig.S1A and 1B).

*The 7-GD virus used for most of the experiments in WSL cells is missing the only confirmed viral component of the extracellular envelope (CD2v/pEP402R) and MGF genes known to be involved in host range and cell tropism.*

As mentioned by the reviewer, 7-GD and ad-7GD have the deletions of EP402R and sixe MGF genes [2]. Ad-7GD was used for most of the experiments due to the fact that we just obtained ad-7GD first when we started this study. To further confirm the function of TMEM239 on ASFV replication, we further used other ASFVs including genotype I SD/DY-I/21, cell-adapted genotype II HLJ/HRB1/21 (ad-HRB1), and wild type genotype II virulent HLJ/18, and our data showed that deletion of TMEM239 affected the infection of all different ASFVs.

[1] Sun E, Huang L, Zhang X, et al. Genotype I African swine fever viruses emerged in domestic pigs in China and caused chronic infection[J]. Emerging microbes & infections, 2021, 10(1): 2183-2193.

[2] Chen W, Zhao D, He X, et al. A seven-gene-deleted African swine fever virus is safe and effective as a live attenuated vaccine in pigs[J]. Science China Life Sciences, 2020, 63: 623-634.

*2.* *The Hernaez paper (reference 37) clearly show that the routes of virus entry in Vero cells and macrophages differ depending on the purity of the virus preparation. The method of virus preparation is missing from the methods and should be included.*

Thank you for your comment. All the virus stocks in this study were purified by using sucrose density gradient centrifugation as previously described [1], and we have added this information in lines 143 - 149 in the revised manuscript.

*In order to interpret their mechanistic data the authors need to define the methods of entry into WSL cells, this could be carried out following some of the methodology described in the Hernaez paper.*

As suggested, we referred to the work of Hernaez et al (reference 37), and we utilized dynasore, a specific inhibitor of the GTPase activity of dynamin, to explore ASFV entry into WSL cells. The results, depicted in lines 496 - 499, Fig. 6C and 6D, demonstrate that dynasore inhibits ASFV internalization, suggesting that ASFV virions enter WSL cells via clathrin-mediated endocytosis (CME).

[1] Wang N, Zhao D, Wang J, Zhang Y, Wang M, Gao Y, et al. Architecture of African swine fever virus and implications for viral assembly. Science. 2019;366(6465):640-4.

*3. This section also needs some data on the effect of TMEM239 deletion on normal endosomal function, are the authors showing a specific effect on ASFV or not?*

Thanks for the reviewer’s comments. We chose Cy3-labeled transferrin to evaluate the impact of TMEM239 deletion on normal endosomal function. The results showed that TMEM239^-/-^ cells exhibited the efficent capacity of transferrin transport comparable to that of wild-type WSL cells, indicating that TMEM239 knockout does not interfere with the normal function of endosomes in lines 512 - 517 (Fig. S4B).

*If the virus isn’t in Rab7+ endosomes, where is it?*

As the reviewer mentioned above, we demonstrated that ASFV enters WSL cells via CME. During CME, virions are internalized with specific receptors via coated membrane invaginations and rapidly form coated vesicles. Subsequently, the vesicle is separated from the plasma membrane through the action of the GTPase dynamin. Ultimately, the vesicle sheds clathrin and matures into early endosomes [1]. We next observed that TMEM239 knockout impedes ASFV entry into early endosomes (Fig.7A - 7C), meanwhile a control group set up as suggested showed that ASFV did not enter Rab7+ endosomes (Fig.S4A). Besides, TMEM239 is involved in small GTPase-mediated signal transduction and transmembrane transport (Fig. 5B and 5C). Summarizing all the evidence, we hypothesized that TMEM239 knockout would block the maturation of ASFV-containing vesicles into early endosomes and the ASFV persists within these vesicles. As the reviewer suggested, the location of ASFV out of endosomes is unclear but valuabe to explore. Since there is still no ideal method to isolate these vesicles, later we will plan to investigate it.

[1] Sousa R, Lafer E M. The role of molecular chaperones in clathrin mediated vesicular trafficking[J]. Frontiers in molecular biosciences, 2015, 2: 26.

*4. The TMEM239-/- data is a strength of the paper, but additional data is required to support the authors conclusions. Any ASFV replication in PBMCs is mostly likely within monocytes and related cells (around 10% of the total cells), therefore the authors need to show that the PBMCs from the TMEM239-/- pigs have a similar number of ASFV susceptible cells and that the same cell types are being infected in their experiments.*

Thanks for the reviewer’s suggestion. As suggested, we analyzed the cellular composition in PBMC from TMEM239^-/-^ pigs and wild-type pigs using flow cytometry. The results showed no significant differences in the proportions of CD45^-^, CD45^+^, Monocyte/Granulocyte, B cells, CD3^+^, CD4^-^CD8^+^, CD4^+^CD8^-^, and CD4^+^CD8^+^ cell populations between TMEM239^-/-^ pigs and wild-type pigs as described in lines 542 - 545 (Fig. S5C - 5E).

As suggested, ASFV predominantly replicates in monocytes, so we analyzed monocytes infected with ASFV by using flow cytometry. Results demonstrated that ASFV predominantly infected monocytes in PBMCs from both TMEM239^-/-^ pigs and wild-type pigs as described in lines 550 - 552 (Fig. S6B - 6D). The infected cell types were consistent in wild-type PBMCs and TMEM239^-/-^ PBMCs. But the proportion of infected monocytes was significantly reduced in PBMC from TMEM239^-/-^ pigs due to the deletion of TMEM239 (Fig. S6B - 6D).

**Minor Issues:**

*1.* *Line 72. ASFV enters through macropinocytosis, a role for micropinocytosis has not been established. Key reference here is Ref 37 which carefully determines that the entry method depends on the cell type and the preparation of the virus stock. This should be included in the introduction.*

Thank you for your comment. As suggested, we have included this part in the Introduction and cited this paper in the revised manuscript (Line: 76 - 78, and Ref: 17).

*2. Line 124 to 126. Where are the* *WSL-adapted viruses described? Information about any changes in the relevant proteins (p72, p12, p30, pE199L, pE248R) should be disclosed or at least discussed, using BA71- Ba71v or Georgia/Vero-adapted Georgia (**Krug et al.) or others as examples. Ideally full genome sequences and comparative analysis of the growth of these viruses in WSL-R and relevant primary macrophages should be included or a citation to this work be provided.*

Thanks for the reviewer’ comments. As suggested, we characterized the genomes of WSL-adapted viruses in the revised manuscript (Lines: 133 - 143). The WSL-adapted strains exhibit no mutations in the relevant proteins (p72, p12, p30, pE199L, pE248R) when compared with their original strains.

In addition, we detected the viral replication kinetics of ASFVs, including the WSL-adapted strains (ad-7GD and ad-HRB1) and the wild-type strain (WT-HLJ18 and WT-SD), and the results showed that all viruses replicated well in PAMs (Fig. S1A and 1B). Besides, the multistep growth curves of the three ASFVs (ad-7GD, ad-HRB1, and WT-SD) in WSL cells were also detected (Fig. 2E and 2F, and Fig. S3A - S3D).

*3.* *Figure 2I. Scale should be similar to that used for 2F.*

As suggested, we have revised the manuscript to ensure uniform scaling for Figure 2.

*Different MOI and virus used in WSL compared to PAMs? (MOI 1 versus MOI 0.1).*

WSL, as a passage-able cell line, exhibits limited support for the replication of naturally isolated wild virulent ASFV strains, such as WT-HLJ18. Consequently, we mainly chosed the WSL cell-adapted strains to perform relevant virus infection experiments in WSL cells. PAMs are target cells of ASFVs. Therefore, we chosed the wild virulent ASFV strain (WT-HLJ18) to perform relevant virus infection experiments on PAMs. In comparison to the WT-HLJ18 strain efficiently and quickly replicating in PAMs, these cell-adapted strains demonstrate slower replication rates in WSL cells. Hence, we selected a higher dose (MOI = 1) for virus replication experiments in WSL cells, as depicted in Figure 2D and 2E. Recognizing the swift replication of WT-HLJ18 on PAMs, we adjusted the viral dose to MOI = 0.1 accordingly.

*4.* *Figure 4. A single cell isn’t particularly convincing without quantitative data (as shown in Figure 7) the rest of the data is fine.*

Thanks for the reviewer’s comments. As suggested, we collected new confocal images for Fig. 4A. The revised figures now encompass multiple cells.

*5.* *Figure 5. One of the most interesting interactions with TMEM239 was Rab14 which the authors found in their original screen. This isn’t mentioned at all and should be included in the discussion, what is known about the interactions between TMEM239 and Rab14?.*

Thanks for the reviewer’s suggestions. LC-MS analysis revealed the potential interaction of TMEM239 with Rab14. Rab14, a member of the RAS oncogene family, is involved in intracellular membrane trafficking [1], the endocytic pathway of cationic substances [2], and switching between GTP/GDP [3]. TMEM239, a sparsely investigated transmembrane (TMEM) protein, potentially involved in GTPase-mediated signal transduction and transmembrane transport (Fig. 5B and 5C). Although both genes share similarities in biological processes, current research predominantly examines TMEM239's influence on ASFV replication.We did not further explore the specific functions between TMEM39 and Rab14. We speculate that TMEM239 and Rab14 may synergistically regulate the above biological processes. As suggested, this part has been addressed in lines 650 - 656 in the Discussion part.

[1] Junutula JR, De Maziére AM, Peden AA, Ervin KE, Advani RJ, van Dijk SM, et al. Rab14 is involved in membrane trafficking between the Golgi complex and endosomes. Molecular biology of the cell. 2004;15(5):2218-29.

[2] Trofimenko E, Homma Y, Fukuda M, Widmann C. The endocytic pathway taken by cationic substances requires Rab14 but not Rab5 and Rab7. Cell reports. 2021;37(5).

[3] Ueno H, Huang X, Tanaka Y, Hirokawa N. KIF16B/Rab14 molecular motor complex is critical for early embryonic development by transporting FGF receptor. Developmental cell. 2011;20(1):60-71.

*6.* *Figure 6. B and C Images are very small and difficult to see. Suggest removing DAPI only panel and enlarging the remaining images.*

Thanks for the reviewer’s suggestion. As suggested, we have reorganized the layout of Figure 6 (original manuscript) and enlarged the figures to enhance visibility in the revised manuscript (Fig. 7A and 7B).

*Localisation of tagged Rab9 and TMEM28 appears to be different. TMEM28 appears endosomal, whereas Rab9 does not and doesn’t match signal in Figure C.*

Hernáez B et al. [1] fused the GFP tag with Rab5 and Rab7 to explore the endocytic transport of ASFV. In current study, fusion of Myc tag with Rab5 may not affect the cellular sublocalization of Rab5. The difference between Rab5 in Figure 6B and Figure 6C could be attributed to the strong signal resulting from overexpression via plasmid transfection in Figure 6B, while the Rab5 in Figure 6C is endogenous to the cell.

*Figure C: A control such as cells incubated at 4oC showing absence of co-localisation between Rab7 and p72 would strengthen the case.*

As suggested, we incorporated a control group (Fig. S4A). And the detailed description has been added in the revised manuscript (lines: 506 - 508).

[1] Hernáez B, Guerra M, Salas M L, et al. African swine fever virus undergoes outer envelope disruption, capsid disassembly and inner envelope fusion before core release from multivesicular endosomes[J]. PLoS pathogens, 2016, 12(4): e1005595.

*7. Figure 7. Use log scales for C and D. Why the difference in replication between MOI 1 of 7-GD and MOI 0.1 of wt?*

Thanks for the reviewer’s comment, we have revised Fig. 7C and 7D (Revised manuscript: Fig. 8C and 8D) to incorporate log scales.

Regarding the question about the difference in replication between MOI = 1 of 7-GD and MOI = 0.1 of wild-type HLJ/18, the wild-type HLJ/18 is a wild-type virulent strain and reaches a viral replication plateau within a short period of infection (Fig.S1A and 1B). Ad-7GD is a WSL-adapted strain that was developed from the seven-gene deletion live attenuated vaccine candidate (HLJ/18-7GD) and replicates slowly in WSL cells. So different MOIs were used for different viruses and cells in this study.

*What cells are actually being infected in these experiments? The authors need to confirm there isn't a difference in the cell populations from the two different animals and then demonstrate there isn't a difference in the type of cells infected.*

As mentioned above, there was no significant difference in the proportions of CD45^-^, CD45^+^, Monocyte/Granulocyte, B cells, CD3^+^, CD4^-^CD8^+^, CD4^+^CD8^-^, and CD4^+^CD8^+^ cell populations between TMEM239^-/-^ pigs and wild-type pigs (Fig. S5C - 5E). Further results demonstrated that ASFV predominantly infected monocytes in PBMC from both TMEM239^-/-^ pigs and wild-type pigs (Fig. S6B). Due to the deletion of TMEM239, the proportion of infected monocytes was significantly reduced in PBMC from TMEM239^-/-^ pigs.

**Response to Reviewer 2**

**Major concerns:**

*1. What was the purpose of using multiple ASFV strains, some being wild-type, some being gene-deleted attenuated viruses? Please provide detailed information on the abbreviations used for the ASFVs.*

Thanks very much for the reviewer’s comments. In this study, we employed WSL cell lines for genome-wide library screening. It's important to note that WSL cells exhibit limited support for the replication of wild-type isolates, such as WT-HLJ18. When we began our genome-wide library screen, we had only one cell-adapted strain, ad-7GD. And ad-7GD could produce fluorescent proteins during replication in WSL cells and induces CPE. This unique attribute made it suitable for the whole-genome library screening. Considering the high variability in gene sequences among ASFV isolates from different genotypes, we further used other ASFVs including genotype I SD/DY-I/21, cell-adapted genotype II HLJ/HRB1/21 (ad-HRB1), and wild type genotype II virulent HLJ/18 to validate the effect of TMEM239 knockout on ASFV replication. And our data showed that deletion of TMEM239 affected the infection of all different ASFVs.

The detailed information on the abbreviations used for ASFV is provided in the revised manuscript (lines: 126 - 143)*.*

*It seems like the ad-7GD virus was used for many experiments described in the manuscript including the screening of the GeCKO WSL library (see Figure 1) and the testing of knock-out cells for ASFV replication (see figure 2). This reviewer believes that Ad-7GD is an attenuated, gene-deleted virus. Were the above-described experiments repeated with a wild-type virus with the same MOI?*

As mentioned before, the WSL cells poorly support wild-type viruses replication, so we did not use the WT-HLJ18 strain for GeCKO WSL library screening, nor did we test knockout cells for ASFV replication. WT-HLJ18 is a wild-type virulent strain and replicates faster. For an example, wild-type pig PBMCs were inoculated with WT-HLJ/18 at MOI = 0.1, resulting in virus copies reaching 10^9^ after 72 h of infection (Fig. 8D), while the ad-7GD was inoculated at MOI = 1, and after 72 h, the virus copies reached 10^7^ (Fig. 8C). Therefore, the doses of inoculation for these two strains of virus are different when conducting virus replication experiments.

*2. Were the ASFVs used in this study sequenced?*

Thanks for the reviewer’s comment. As suggested, we conducted deep sequencing and assembled the viral genomes of the other two WSL-adapted strains (ad-HRB1 and ad-7GD). The whole genome sequences of WT-SD and WT-HLJ18 strains were previously reported [1, 2]. And the detailed description has been shown in lines lines 126-143 in the revised manuscript.

*How were these viruses amplified and their respective titer obtained?*

As suggested, the methods used for preparing the viral stocks have been shown in the revised manuscript (lines 143 - 149). To determine the viral titers of ad-HRB1, ad-7GD, and WT-SD, the virus stocks underwent stepwise gradient dilution followed by inoculation onto WSL cells for approximately 96 h. The titers of ad-HRB1 and WT-SD were measured through indirect immunofluorescence (Anti-p72). For ad-7GD, which produces fluorescent proteins during replication, positive wells were directly observed and recorded. Regarding WT-HLJ18, the 50% HAD dose (HAD_50_) for this strain was measured as described in lines 335 - 343 of the revised manuscript.

[1] Sun E, Huang L, Zhang X, et al. Genotype I African swine fever viruses emerged in domestic pigs in China and caused chronic infection[J]. Emerging microbes & infections, 2021, 10(1): 2183-2193.

[2] Zhao D, Liu R, Zhang X, et al. Replication and virulence in pigs of the first African swine fever virus isolated in China[J]. Emerging microbes & infections, 2019, 8(1): 438-447.

*3. The authors custom designed approx. 186 000 sgRNA targeting more than 20,000 swine genes. They have not provided any of the sgRNA sequences, names of target genes or number of sgRNAs per gene. This information needs to be provided in supplementary files. It is not possible for this reviewer to evaluate the present manuscript especially considering the claim of the authors that that their swine library design is better than the previously published ones.*

Thanks for the reviewer’s suggestion. We provided comprehensive information about the swine genome-wide CRISPR knockout screen sgRNA library in Table S1, including sgRNA sequences and names of target genes.

*4. The method to develop* *knock-out WSL cells is not described in detail.*

Thanks for the reviewer’s suggestion. We have included comprehensive information on the sgRNA sequences (Table S3) and the associated methods (Lines: 232 - 244) utilized for the knock-out of both the TMEM239 and Rab14 genes in the revised manuscript.

*5. The authors did not use appropriate negative control WSL cells for evaluation of their candidate genes.* *Non-target control sgRNA WSL cells must be used as a negative control instead of wild-type WSL cells.*

Thanks for the reviewer’s comments. Prior to conducting our genome-wide library screening, we conducted a preliminary assessment to ascertain the impact of Cas9 expression cassette insertion and lentivirus transduction on viral replication in WSL cells. Our findings, as presented in Fig. S1C and 1D, demonstrated that ad-7GD replicated similarly in both WSL cells and WSL-Cas9 cells transduced with lentivirus carrying gRNA targeting GGTA1. This preliminary experiment provided crucial evidence that the insertion of the Cas9 expression cassette and lentiviral transduction did not significantly affect viral replication in WSL cells.

As suggested, we employed lentiviral transfection to establish a cell line carrying a non-target control sgRNA termed WSL-NC-sgRNA. Subsequently, ad-7GD was inoculated separately on WSL cells and WSL-NC-sgRNA cells. The results, depicted in Fig. 2C and 2D, demonstrated no significant difference in virus replication between the two cell lines. These findings support that both WSL cells and non-target control sgRNA WSL cells can serve as effective negative controls when validating candidate genes.

*6. The authors have not provided data on the list of genes, number of sgRNAs enriched or ranking of the genes which were enriched after ASFV replication – only TMEM239, SLA-DMB and Rab14 are mentioned (Lines 289-290; Fig 1H). Please provide the list of genes enriched in the selection process. Please also include gene ranking and the number of sgRNAs per gene in a supplementary file (see lines 176- 178).*

Thanks for the reviewer’s comment. We have added information on all enriched genes and their respective rankings in Table S4 as shown in lines 380-381 in the revised manuscript.

*7. There is no information about the replicates of GeCKO WSL cells which were generated. Usually, more than 2 replicates are required to reduce false discovery rate and increase the reliability of data.*

Thanks for the reviewer’s suggestion. In our experimental procedures, subsequent·to the infection of WSL-Cas9 cells with the·GeCKO lentiviral library, the number of GeCKO·WSL cells increased 3-4-fold during·the two-week puromycin selection. Subsequently, a portion of cells (~1.8 X 10^8^ cells) were taken for sequencing to elucidatethe sgRNA·content within the GeCKO WSL cells. Simultaneously, another portion (~1.8 X 10^8^ cells) was allocated for the ASFVscreening experiment. The remaining cells were frozen and planned to be used for repeat screening. And the detailed description has been added in the revised manuscript (lines: 219 - 223). However, when the genes (TMEM239 and Rab14) obtained in the first screening were experimentally verified to be involved in the replication process of ASFV, we did not repeat the screening. In addition, the top candidate gene (SLA-DMB) we screened has also been reported in published paper [1]. We think that our screening data should also be reliable.

[1] Pannhorst K, Carlson J, Hölper JE, Grey F, Baillie JK, Höper D, et al. The non-classical major histocompatibility complex II protein SLA-DM is crucial for African swine fever virus replication. Scientific Reports. 2023;13(1):10342.

*8. The authors apparently have access to TMEM239-/- knock-out pigs. What is the phenotype of these pigs and why were they not used for AFV infection studies?*

Thanks for the reviewer’s comments. All TMEM239^-/-^ knockout piglets were in good health after birth. According to the normal immunization program, these piglets were vaccinated with vaccinations include swine fever virus vaccine, porcine reproductive and respiratory syndrome virus (PRRSV) vaccine and porcine circovirus vaccine. However, during subsequent feeding, one of the piglets (#355-2) displayed symptoms such as diarrhea, swollen joints, and lethargy. It was further identified that the piglet was infected with PRRSV. Additionally, we randomly selected three piglets from the remaining healthy piglets, extracted peripheral blood, and analyzed the composition of peripheral blood lymphocytes using flow cytometry analysis. The results showed that CD45^+^, CD3^+^, CD4^+^CD8^-^, CD4^-^CD8^+^, CD4^+^CD8^+^, B cells, and monocytes/granulocytes in peripheral blood lymphocytes of TMEM239^-/-^ knockout piglets were similar to those of wild-type piglets (Fig.S5C - S5E).

Due to the small size of the TMEM239^-/-^ pig population and the limited experimental space in our ABSL3 laboratory, currently we did not perform ASFV infection experiment using TMEM239^-/-^ pigs. Later we have the plan to conduct validation experiments after the population has been expanded to allow for a more comprehensive investigation.

*9. Introduction, lines 89-96: Please update your introduction section with current literature on host factors already identified for ASFV.*

As suggested, we have integrated relevant literature on host factors that have already been identified for ASFV in lines 84 - 92 in the revised manuscript.

*10. Methods, lines 133-134: Please provide details about the production and specificity of the monoclonal antibodies.*

Thanks for the reviewer’s suggestions. The antibodies targeting ASFV proteins (p30, p54, and p72) were developed in our laboratory. Production and specificity details of mouse monoclonal anti-p54 and p-30 has been descripted in privious studies [1, 2]. And the detailed description has been added in the revised manuscript (lines 159 - 161).

The p72 polyclonal antibody was also developed in our laboratory and the production process is described in line 161 - 164 of the revised manuscript. As for p72 antibody specificity, In the newly revised Fig. 4A, anti-p72 antibody was used to evaluate the expression of viral p72 protein, showing fluorescence only in virus-infected cells. These images support the specificity of the p72 antibody.

[1] Tesfagaber W, Wang L, Tsegay G, Hagoss YT, Zhang Z, Zhang J, et al. Characterization of Anti-p54 Monoclonal Antibodies and Their Potential Use for African Swine Fever Virus Diagnosis. Pathogens. 2021;10(2). Epub 2021/02/11. doi: 10.3390/pathogens10020178. PubMed PMID: 33562314; PubMed Central PMCID: PMCPMC7915713.

[2] YU Sh, SHAN Zm, YANG Jj, LIU Yn, WU Cd, ZHANG Zj, et al. Identification of two novel linear epitopes on the p30 protein of African swine fever virus. Journal of Integrative Agriculture. 2023;22(6):1945-9.

*11. Methods, lines 244-253: What was the purpose for generation* *polyclonal knock-out cell lines? Monoclonal TMEM239 knockout cells were already established.*

Thanks for the reviewer’s comments. The off-target effect is an unavoidable drawback of the current CRISPR/Cas9 gene editing technology. To exclude potential off-target effects of the sgRNA used to establish monoclonal TMEM239 knockout cells, we employed three additional sgRNAs targeting TMEM239. This strategy allowed us to generate polyclonal TMEM239 knock-out cell lines, thereby providing a means to exclude off-target effects and ensure the robustness of our findings. The revised manuscript now includes this clarification in lines 398 - 399.

**Minor Issues**

*1. Line 115-123: Were antibiotics/antimycotics not included in the medium formulation?*

Thanks for the reviewer’s comment. Actually we added antibiotics to the culture medium. We included the additional details regarding the antibiotic supplementation in the medium in lines 116 - 125 in the revised manuscript.

*2. Line 128-134: Can you please provide the catalogue numbers for the antibodies used in the study.*

As suggested, we have now incorporated the respective catalog numbers for the antibodies used in the study in lines 151 - 158 in the revised manuscript.

*3. Line 136: How was the cDNA for the TMEM239 and Rab5A genes obtained? Please provide details.*

As suggested, we added the detailed information on how to obtain the cDNAs in lines 166 - 168 in the revised manuscript as following: The cDNA sequences of the porcine TMEM239 gene and Rab5A gene to Genscript (NanJing, China) for sequence synthesis.

*4. Line 165: Please provide details of how the WSL-Cas9 was constructed. What was the source of the vector?*

As suggested, we have included detailed information on the construction of the WSL-Cas9 cell line in the revised manuscript (Lines: 199 - 211). And the vector used in this process was constucted by us, leveraging the PUC57 vector as a backbone.

*5. Line 189: What culture medium was used?*

Thanks for the reviewer’s comment. We employed Dulbecco's modified Eagle's medium (DMEM) supplemented with 2% fetal bovine serum (FBS; Gibco, USA) for the multi-step growth curve of the virus. We have incorporated a detailed description in the revised manuscript (line 248).

*6. Lines 191-192: How did you determine the viral titer in the* *supernatants?*

Thanks for the reviewer’s comment. The viral titer in the supernatants was measured by using TCID_50_ or HAD_50_ as mentioned above. In brief, the supernatants underwent stepwise gradient dilution followed by inoculation onto WSL cells for approximately 96 h. The titers of ad-HRB1 and WT-SD were measured through indirect immunofluorescence (Anti-p72) for TCID_50_. For ad-7GD, which produces fluorescent proteins during replication, positive wells were directly observed and recorded for HAD_50_.

*7. Lines 194-195: Please provide catalogue numbers for NTC and TMEM239 siRNAs.*

Thanks for the reviewer’s suggestion. we have included the information on the companies that synthesized the siRNAs in lines 255 - 257. Additionally, the sequences of the siRNAs can be found in Table S3 in the revised manuscript.

*8. Line 202: What virus is ad-HRB1? What is the purpose of using this and not other ASF viruses for these experiments?*

Thanks for the reviewer’s comments. As mentioned in lines 133 - 140 in the revised manuscript, ad-HRB1 is a genotype II strain of WSL-adapted ASFV, derived from naturally occurring lower virulent ASFV HLJ/HRB1/20 [1]. When we started this study, we just obtained WSL-adapted genotype II gene-deleted HLJ/18-7GD (ad-7GD) first. So ad-7GD was used for CRISPR/Cas9 screen. Later to further confirm the function of TMEM239 on ASFV replication, we used other ASFVs including genotype I SD/DY-I/21, ad-HRB1, and wild type genotype II virulent HLJ/18, and our data showed that deletion of TMEM239 affected the infection of all different ASFVs. Due to the fact that genotype II ASFV is widely prevalent in China, and ad-7GD has more gene deletion than ad-HRB1, we used ad-HRB1 for the experiments in Fig. 3 and in the subsequent experiments.

[1] Sun E, Zhang Z, Wang Z, et al. Emergence and prevalence of naturally occurring lower virulent African swine fever viruses in domestic pigs in China in 2020. Sci China Life Sci. 2021 May;64(5):752-765. doi: 10.1007/s11427-021-1904-4. PubMed PMID: 33655434.

*9. Lines 216 and 226: Please provide details of how the Flag-tagged TMEM239 and myc-tagged Rab5A plasmids were constructed.*

Thanks for the reviewer’s suggestion. The detailed information for the construction of Flag-tagged TMEM239 and myc-tagged Rab5A plasmids was added in lines 166 - 170 in the revised manuscript.

*10. Line 235: This reviewer is not sure whether the wild-type WSL cell is an appropriate control. Why did the authors not use non-target sgRNA control WSL cells as the appropriate controls?*

Thanks for the reviewer’s suggestion. As suggested, we used non-target control sgRNA WSL cells as negative control when validating candidate genes as described in lines 394 - 396, and found that the results were similar with that using wild-type WSL cell.

*11. Line 239: What is the name of the primary antibody used here?*

Thanks for the reviewer’s suggestion. The primary antibody used here specifically targets the capsid protein p72 of ASFV. This antibody was produced in our laboratory as described in lines 160 - 164.

*12. Line 261: How was the Cas-9 cassette inserted into Rosa26 locus? This is not described in the Methods section.*

Thanks for the reviewer’ comment. We have added the information for Cas-9 cassette inserting into Rosa26 locus in lines 200 - 211 in the revised manuscript.

*Also, why Rosa26 locus was selected?*

Many studies have proved that the Rosa26 locus is a “safe harbor” and is conserved in multiple species, including mice [1], human [2], rats [3] and pigs [4]. In addition, the sequence of the pig Rosa26 locus has been completely characterized [5]. Rosa26 gene of porcine has been reported to be suitable for driving exogenous gene expression in a high and stable manner by avoiding DNA methylation [6]. Therefore, we selected Rosa26 locus for the insertion of the Cas9 expression cassette in order to basically not affect the phenotype of WSL cells and to more possibly obtain WSL-Cas9 with high cleavage activity.

[1] Chu, V. T. et al. Increasing the efficiency of homology-directed repair for CRISPR-Cas9-induced precise gene editing in mammalian cells. Nat Biotechnol 33, 543–8 (2015).

[2] Irion, S.et al. Identification and targeting of the ROSA26 locus in human embryonic stem cells. Nat Biotechnol 25, 1477–82 (2007).

[3] Kobayashi, T. et al. Identification of rat Rosa26 locus enables generation of knock-in rat lines ubiquitously expressing tdTomato. Stem Cells Dev 21, 2981–6 (2012).

[4] Li, X. P. et al. Rosa26-targeted swine models for stable gene over-expression and Cre-mediated lineage tracing. Cell Research 24, 501–504 (2014).

[5] Li X, Yang Y, Bu L, et al. Rosa26-targeted swine models for stable gene over-expression and Cre-mediated lineage tracing[J]. Cell research, 2014, 24(4): 501-504.

[6] Kong, Q. et al. Rosa26 locus supports tissue-specific promoter driving transgene expression specifically in pig. PLoS One 9, e107945 (2014).

*13. Line 285: Did the ad-7GD ASFV causes CPE at 1 MOI?*

Thanks for the reviewer’s comment. When WSL cells are infected with ad-7GD at an MOI of 1, the cells exhibit distinct cytopathic effects (CPE) within approximately 4 days. This phenomenon is visually captured in Figure A:

**Figure legends: Ad-7GD caused CPE in WSL cells at 1 MOI. (A)** WSL cells were infected with ad-7GD (MOI = 1) for 4 days.

*14. Line 299: Figure 2A and 2B shows gene transcription (mRNA) analyses. Was quantitative PCR or RT-PCR used. Also, confirm in legend of Figure 2.*

Thanks for the reviewer’s comments. In Figure 2A and 2B, we conducted RT-qPCR, respectively, to quantify the transcript levels of these genes in TMEM239^-/-^ and Rab14^-/-^ cell lines. We have revised this part in line 959 in the revised legend of Figure 2.

*15. Lines 306-308: Was the KO of the TMEM239 gene in the polyclonal KO cell lines confirmed?*

Thanks for the reviewer’s comment. To ascertain the knockout (KO) status of the TMEM239 gene, we used RT-qPCR to detect the level of TMEM239 transcript in the polyclonal KO cell lines, and the results showed that TMEM239 transcript was significantly decreased in the polyclonal KO cell lines as presented in Fig. S2C.

*16. Line 313: This reviewer does not agree with the conclusion of the authors that there is a plateau of virus replication after 168 hpi (see Figure 1F). The virus titer is higher at 196 hpi than at 168 hpi.*

Thanks for the reviewer’s comment. We have modifed this part in lines 406 - 408 in the revised manuscript as following “Viral replication in WSL cells increased significantly with prolonged infection, whereas viral replication in TMEM239^-/-^ cells remained at a consistently lower level (Figures 2E and 2F)”.

*17. Line 422: “SCNT”. Please define.*

Thanks for the reviewer’s suggestion. We included the definition for the acronym "SCNT" in lines 530 - 531 in the revised manuscript.

*18. Line 425: How can a heterozygote piglet be born from a embryo obtained from a homozygous TMEM239 KO cell line?*

Thanks for the reviewer’s comment. Somatic cell nuclear transfer (SCNT) stands as an established technology in genetic breeding with widespread applications. The observed occurrence of heterozygous mutant piglets derived from biallelic deletion donors in this study has also been reported in other studies. Yin Y et al. utilized MC3R biallelic deletion PEF cells as donor cells, resulting in the birth of 6 piglets, among which only 2 exhibited the desired MC3R gene deletion [1].

Theoretically, the genotype of the piglet should precisely mirror that of the donor cells. However, one potential condition leadinging to the production of heterozygous mutants might be the presence of a small fraction of wild-type cells or cells with other genotypes within the biallelic deletion donor cell population used in our work. The genotype identification of gene-edited PEF cells was performed by extracting DNA from part of the cells (< 100 cells), amplifying the bands through conventional PCR technology, and then performing Sanger sequencing. If biallelic deletion PEF cells contain a small population of cells with different genotypes, the true genotype of the PEF cells may be masked using this identification method. In our subsequent SCNT, we need tens of thousands of cells. If the above conditions are present, cells with different genotypes will result in piglets that may exhibit a genotype that is inconsistent with the donor cells. Therefore, it is very necessary to genotype the piglets.

[1] Yin Y, Hao H, Xu X, et al. Generation of an MC3R knock-out pig by CRSPR/Cas9 combined with somatic cell nuclear transfer (SCNT) technology[J]. Lipids in health and disease, 2019, 18: 1-8.

*19. Line 426: What are “suboptimal physical conditions”. Please explain. Are these pigs sick, not growing, dying?*

Thanks for the reviewer’s comment. In original manuscript, we only described TMEM239^-/-^ piglets as "Given the limited number and suboptimal physical condition of the piglets.". Actually all TMEM239^-/-^ knockout piglets were in good health since birth. According to the normal immunization program, these piglets were inoculated with different commercial vaccines including swine fever virus vaccine, porcine reproductive and respiratory syndrome virus (PRRSV) vaccine, and porcine circovirus vaccine. However, during subsequent feeding, one of the piglets (#355-2) displayed symptoms such as diarrhea, swollen joints, and lethargy. It was further identified that the piglet was infected with PRRSV. In order to avoid ambiguity, we revised this sentence in line 538 in the revised manuscript. And the detailed description has been shown in lines lines 534 - 540 in the revised manuscript.

*20. Line 437: These results are obtained in pig PBMCs not in pigs.*

Thanks for the reviewer’s suggestion. We have revised it in line 557.

*21. Line 459: What does “greater storage capacity” mean? Please explain.*

Thanks for the reviewer’s comment. Here we want to state that our library encompasses a larger number of sgRNAs and more sgRNAs targeting each gene than SsCRISPRko.v1. Acturally SsCRISPRko.v1 library comprises 83,381 specific sgRNAs targeting 20,598 porcine genes along with 1001 non-targeting controls. In our study, the employed sgRNA library consists of 186,510 specific sgRNAs targeting 20,580 porcine genes and 1000 non-targeting controls. To avoid confusing this part, we revised in lines 576 - 578 in the revised manuscript.

*22. Line: 497: “CME”. Please define.*

Thanks for the reviewer’s suggestion. We have revised it in line 616.

*23. Line 529: Citation (60) belongs to the previous sentence.*

Thanks for the reviewer’s suggestion. We have revised it in line 668, ref: 72.

**Response to Reviewer 3**

**Major concerns:**

*1. The authors describe TMEM239 as crucial to ASFV infection and replication, suggesting an essential role. However the virus is still able to infect cells and replicate, although replication is significantly reduced. While the replication data in figure 2 shows a 2 log reduction in ASFV replicatino based on TCID50 assays, similar experiments using other strains of ASFV shown in FigS2 indicate far less impressive reductions, in one case less than a log. The authors do not address this discrepancy.*

Thanks very much for the reviewer’s comments. As the reviewer mentioned, the three strains (ad-7GD, ad-HRB1, and WT-SD) showed different levels of reduction in replication in TMEM239^-/-^ WSL cells. We assume that this is mainly related to the differences in the genomes of the different strains, and addressed this discrepancy in lines 638 - 650 in Discussion part. The identity genome between ad-7GD and ad-HRB1 was 99.56%; and SD/DY-I/21 showed 97.25% and 97.48% genomic identity with ad-7GD and ad-HRB1, respectively. Fig. 2 illustrates 2-log reduction in ASFV replication for ad-7GD on TMEM239^-/-^ WSL cells based on TCID50 assays, and ad-HRB1 displayed more than 1-log reduction, while SD/DY-I/21 showed less than 1-log reduction in ASFV replication (Fig. S3), consistent with the genomic differences among strains.

*2. The authors use siRNAs to KD TMEM239 in PAM cells. Good practice requires the use of multiple siRNAs to avoid misinterpretation of off target effects. The authors employed three siRNAs, however only reported the results from a single siRNA, despite two siRNAs having the same KD efficiency.*

Thanks very much for the reviewer’s suggestion. We repeated the siRNA interference experiments to evaluate the effects of all three siRNAs used for TMEM239 knockdown in PAM cells on viral replication, and included all data of three siRNAs in the revised manuscript (Fig. 2H and 2I).

*The phenotypic effect is also very slight. Again this was not addressed.*

As the reviewer mentioned, the inhibition phenotype of viral replication was not as obvious as when TMEM239 is knocked out in WSL cells, but this result is stable and significant across repeated experiments as shown in lines 416 - 423. Two potential factors may contribute to the slight phenotype. First, the efficacy of siRNA interference in reducing TMEM239 expression is not as potent as the complete knockout observed in WSL cell experiments. The second factor is the difference in virus strains. As mentioned above, different ASFV strains showed different levels of replication reduction in TMEM239^-/-^ WSL cells. Here, we employed the wildtype virulent strain HLJ/18 and its susceptible primary PAMs for the siRNA interference experiments in order to confirm the effect of TMEM239 on ASFV replication, because we performed CRISPR/Cas9 using WSL-adapted ASFV and WSL cells not susceptible to wildtype ASFVs.

*3. Adsorption assays were used to determine which stage TMEM239 impacts virus infection. The authors conclude that KO of TMEM239 does not impact adsorption, however, without a positive control it is difficult to interpret a negative result.*

Thanks for the reviewer’s comments. Regarding the positive control, we reviewed the literature and did not find specific reports on host genes or treatments known to inhibit ASFV adsorption, hence the challenge in incorporating a traditional positive control. In our experimental, we conducted a comparative analysis between TMEM239^-/-^ cells and wild-type cells, repeating the experiments independently for three times to ensure robustness. The consistent outcome across multiple trials revealed no discernible difference in the adsorption capacity of ASFV to TMEM239^-/-^ cells compared to wild-type cells. Based on these results, we concluded that the knockout of TMEM239 does not impact the adsorption stage of ASFV. In other studies, such as those investigating host factors required for avian influenza virus and rabies virus replication, only wild-type cells were used as controls to explore whether down-regulation of host factors affects these viruses adsorption [1, 2].

[1] Song Y, Huang H, Hu Y, et al. A genome-wide CRISPR/Cas9 gene knockout screen identifies immunoglobulin superfamily DCC subclass member 4 as a key host factor that promotes influenza virus endocytosis[J]. PLoS Pathogens, 2021, 17(12): e1010141.

[2] Wang X, Wen Z, Cao H, et al. Transferrin receptor protein 1 cooperates with mGluR2 to mediate the internalization of rabies virus and SARS-CoV-2[J]. Journal of Virology, 2023, 97(2): e01611-22.

*4. In figure 4 the authors suggest that viral expression levels of early and late genes suggest TMEM239 plays a role at early stages of infection. However, the impact of TMEM239 KO was far more prominent for the late gene than the early gene, suggesting a role subsequent to early events.*

Thanks for the reviewer’s comments. Our current data in Figure 4 supported the effect of TMEM239 on the early stage of ASFV infection at least, which could not demonstrate Whether TMEM239 plays a role at the late stage of ASFV infection. As the reviewer mentioned, the early gene *CP204L* (p30) was affected already at the beginning of its expression in TMEM239 knockout cells, although the late gene B646L (p72) is more affected at the late stage. One of the reasons is that this phenomenon is similar to “signal amplification”. During the initial stages of ASFV infection, viral genes expression are extremely low, and the difference in viral gene expression between wild-type cells and TMEM239 knockout cells may be slight. As the infection progresses, viral late genes begin to be expressed in large quantities from 6 h post-infection, so that differences caused at early stage induced more aggravated difference at the late stage.

*5. Figure 5 reports associations of TMEM239 by proteomics. However, this was applied in uninfected cells, so is less relevant when determining the its role in ASFV infection. I appreciate that such experiments are difficult with ASFV, but the limitations of the approach should be acknowledged.*

Thanks for the reviewer’s comment. Limited research on TMEM239 prompted us to perform a proteomics-based gene function prediction. In initial experiments, we want to utilize pull-down assays to identify host proteins and viral proteins that interact with TMEM239. However, the LC-MS analysis of the pCAGGS-TMEM239-Flag + ASFV group did not detect ASFV proteins. Consequently, we only present the mass spectrometry data from Fig. 5A in the original manuscript. When we examined the results of LC-MS analysis of the pCAGGS-TMEM239-Flag with ASFV infection, we found that Rab5 is also a binding partner of TMEM239. In addition, as suggested, we included the data on the pCAGGS-TMEM239-Flag with ASFV infection in the revised Fig. 5A and Table S6.

*I was also left unconvinced by the confocal data suggesting loss of the co-localisation of p72 and RAB5C. Even if there is a suggestion that this occurs additional supporting data, such as direct interaction of viral proteins with TMEM239 would be required before the authors can conclude that the host factor is required for viral entry into early endosomes.*

Thanks for the reviewer’s comment. As suggested, we selected eight viral proteins to identify whether they interact with the TMEM239 by using co-immunoprecipitation assays as described in lines 500 - 502 and Fig. 6E. The results revealed that viral protein pE248R was a TMEM239 binding partner, which was proved to be involved in early post-entry event of ASFV [1].

[1] Rodríguez I, Nogal M L, Redrejo-Rodríguez M, et al. The African swine fever virus virion membrane protein pE248R is required for virus infectivity and an early postentry event[J]. Journal of virology, 2009, 83(23): 12290-12300.

*6. Finally the authors test PAMs derived from transgenic TMEM239 KO piglets and show reduced virus replication. However a different assay was used here to measure virus replication - hemadsorption, or HAD50. The assay is not defined in the text or described in the methods. This is a crucial experiment to demonstrate the phenotype observed in WSL cells occurs in the more relevant PAMs.*

Thanks for the reviewer’s suggestion. We isolated the peripheral blood mononuclear cells (PBMCs) from transgenic TMEM239 KO piglets, and infected PBMCs from TMEM239 KO piglets and wildtype piglets using ASFV. Viral titers in the cell supernatants were measured by using HAD_50_. As suggested, we have added the details of the HAD_50_ assay in lines 335 - 343 in the revised manuscript.

*However, in FigS3C, while fluorescence intensity is reduced in the KO cells, many of the cells are still clearly infected. This again is not addressed by the authors.*

We evaluated ASFV replication in primary PBMCs derived from TMEM239 KO piglets and wildtype piglets. As we know, primary PBMCs may include different kinds of ASFV susceptible cells such as monocyte, macrophage, and granulocyte with different differentiation stages. Monocytes are also generally divided into three main groups: classical, non-classical, and intermediate [1], and macrophage can be divided into M1 macrophages (CAM) or M2 macrophages (AAM) [2]. Maybe TMEM239 supports ASFV replication in different kinds of cells with a celltype dependent manner. As suggested, this part has been addressed in lines 678 - 682 in the Discussion part.

*In addition, KO of TMEM239 has significant impacts on the health of the piglets. PAMs derived from these pigs could have defects that impact ASFV replication indirectly. Rescuing virus replication by restoring TMEM239 expression in these cells would be required to rule this out. Again, I understand that such experiments may be challenging due to the poor health of the piglets.*

Thanks so much for the reviewer’s suggestion. In our original manuscript, we mentioned that "Given the limited number and suboptimal physical condition of the piglets.", which may be confusing. Actually all TMEM239^-/-^ knockout piglets were in good health since birth. According to the normal immunization program, these piglets were inoculated with different commercial vaccines including swine fever virus vaccine, porcine reproductive and respiratory syndrome virus (PRRSV) vaccine, and porcine circovirus vaccine. However, during subsequent feeding, one of the piglets (#355-2) displayed symptoms such as diarrhea, swollen joints, and lethargy. It was further identified that the piglet was infected with PRRSV. In order to avoid ambiguity, we revised this sentence in line 540 in the revised manuscript. And the detailed description has been shown in lines lines 534 - 540 in the revised manuscript. Furthermore, we analyzed the cellular composition in PBMC from TMEM239^-/-^ pigs and wild-type pigs using flow cytometry. The results revealed no significant differences in the proportions of CD45^-^, CD45^+^, Monocyte/Granulocyte, B cells, CD3^+^, CD4^-^CD8^+^, CD4^+^CD8^-^, and CD4^+^CD8^+^ cell populations between TMEM239^-/-^ pigs and wild-type pigs (Fig. S5C - 5E).

As suggested, we attempted to transfect the TMEM239-expressing plasmid into PBMCs of TMEM239^-/-^ pigs to restore the TMEM239 expression by using electrotransfection. However, TMEM239 expression was undetectable in the electrotransfected cells via western blot analysis, and we did not find the recovery of ASFV replication in PBMCs of TMEM239^-/-^ pigs after restoring the TMEM239 expression. This may be due to the difficulty of transfecting PBMCs. In the future, we will plan to restore TMEM239 expression in TMEM239^-/-^ pigs to identify whether ASFV replication can be rescued or not.

[1] Kapellos T S, Bonaguro L, Gemünd I, et al. Human monocyte subsets and phenotypes in major chronic inflammatory diseases[J]. Frontiers in immunology, 2019, 10: 482347.

[2] Tardito S, Martinelli G, Soldano S, et al. Macrophage M1/M2 polarization and rheumatoid arthritis: a systematic review[J]. Autoimmunity reviews, 2019, 18(11): 102397.

**Minor Issues:**

*1. Lines 78-84. Not clear on the relevance of describing the roel of these viral genes to this paper?*

Thanks for the reviewer’s comment. We have revised this part in lines 67 - 82.

*2. Given this paper uses a CRISPR screen to identify host factors important for ASFV replication, a more detailed discussion of Pannhorst et al is warranted in the introduction.*

Thanks for the reviewer’s suggestion. As suggested, we have incorporated the work conducted by Pannhorst et al. into the Introduction section in lines 96 - 98 in the revised manuscript.

*3. Bit confused by figure two. The authors describe generating monoclonal knockout cells and showing a phenotypic impact on virus replication. Then describe generation of polyclonal KO cells?*

Thanks for the reviewer’s comment. The off-target effect is an unavoidable drawback of the current CRISPR/Cas9 gene editing technology. To exclude potential off-target effects of the sgRNA used to establish monoclonal TMEM239 knockout cells, we employed three additional sgRNAs targeting TMEM239. This strategy allowed us to generate polyclonal TMEM239 knock-out cell lines, thereby providing a means to exclude off-target effects and ensure the robustness of our findings. To clarify this part, we revised it in lines 398 - 399.

*4. Figure 7 C and D, not clear why it was necessary to break the scale. Interpretation would be easier with a normal uninterrupted scale.*

Thanks for the reviewer’s suggestion. We have updated it in the new Fig. 8C and 8D in the revised manuscript.
