# Supplementary material for: A genome-wide CRISPR/Cas9 knockout screen identifies TMEM239 as an important host factor in facilitating African swine fever virus entry into early endosomes
Source: PLoS Pathog. 2024 Jul 18;20(7):e1012256. doi: 10.1371/journal.ppat.1012256 (PMC11288436; doi:10.1371/journal.ppat.1012256)
Supplement: Attachment — Submitted filename: Response letter to Reviewers.docx [file ppat.1012256.s015.docx]

**Response letter to Reviewers’ comments (PPATHOGENS-D-23-02109R1)**

**Point-by-point responses to the comments of the Editor and reviewers and a list of changes are:**

(The comments of the Editor and Reviewers are in italics and blue color, which are followed by our responses. All main revisions are highlighted using yellow color in the revised manuscript.)

**Response to Editors amd Reviewers.**

*1. TMEM239 knockdown or knockout reduces replication of different ASFV strains by a relatively small amount, one or two logs maximum. Therefore, it is not accurate to describe TMEM293 as a “crucial host factor in facilitating ASFV entry into early endosomes”. The title should be reworded to better represent the results. For example, use the word “important” rather than “crucial”. This applies to other sections of the manuscript which describes TMEM293 as a crucial host factor for example the heading on line 283, heading of figure S2.*

Thanks for the reviewer’ suggestion. We have revised the title of this manuscript as “A genome-wide CRISPR/Cas9 knockout screen identifies TMEM239 as an important host factor in facilitating African Swine Fever Virus entry into early endosomes”, and also replaced the word “crucial” or “key” with “important” in lines 374, 555, 581, 682, 683, and 1093 in the whole manuscript.

*2. A major point raised by the reviewers was the inadequate description and rationale for use of the different ASFV strains used in the study. Explanation has been provided in Methods Cells and Viruses section of the characteristics of these strains including genes deleted from cell adapted or attenuated strains, how they were derived and cell tropism. Including a table or diagram to show the parental strain, how they were derived and which genes are deleted from different strains compared to the highly virulent wild type strain would help understanding. Accession numbers of full genome sequences could be included in the table.*

Thanks so much for the reviewer’s suggestion. We have made a supplementary table (Table S1) as described in lines 135 - 136 , which includes the source of the strains used in this study, the accession number, the number of passages of the adapted strain, and the deleted genes.

*3. A further point raised by reviewers was that many experiments were carried out using cell culture adapted or gene-deleted viruses. Thus it is possible that genome differences between isolates may account for variation in replication levels and may not necessarily be associated TMEM293 knockout. Although this is mentioned in the revised Discussion (starting line 638), it is important to further discuss the potential limitations of the study in regard to the use of attenuated viruses.*

Thanks for the reviewer’s comments. As there is currently no cell line capable of effectively supporting the replication of all wild-type ASFVs, we employed gene-deleted and cell-adapted strains which could efficiently replicate in WSL cells to perform the GeCKO screen and investigate the impact of TMEM239 gene knockout on ASFV replication. To further reflect the importance of the host factor TMEM239 for replication of the wild-type ASFV, we knocked down the expression of the TMEM239 gene in PAM by siRNA interference. The results showed that knockdown of TMEM239 significantly affected wild-type ASFV replication in PAM as described in lines 404 - 414, which suggests the close relativeness of TMEM239 with ASFV replication.

As mentioned by the reviewer, ad-7GD, ad-HRB1, and WT-SD have different replication abilities in wild-type WSL cell line, which was also confirmed by the viral growth curves of the three strains (Fig 2E, 2F, and Fig S3). The reason for this phenomenon may be the large genome differences among the three strains as described in lines 634 - 637. In our experiments to validate the effect of TMEM239 gene deletion on the replication ability of different strains, we set up wild-type WSL cells as a control group. For example, when we verified the effect of TMEM239 gene deletion on the replication of the WT-SD strain, the WT-SD strain was used to infect TMEM239^-/-^ cells and wild-type WSL cells, respectively. And we then compared the replication levels of the WT-SD strain in wild-type and TMEM239^-/-^ cells to determine the effect of the TMEM239 gene. All results indicated that the replication ability of the three strains in TMEM239^-/-^ cells was lower compared to that in the corresponding wild-type WSL cells as described in lines 395 - 403. In conclusion, deletion of TMEM239 significantly affects ASFV replication.

As concerned by the reviewer, there are some limitations when we used gene-deleted and cell-adapted ASFV strains to perform the GeCKO screen in WSL cells, because utilizing cell-adapted strains in conjunction with cell lines may not provide an ideal representation of wild-type ASFV replication in its susceptible PAM. So all the results should be further validated by using wild-type ASFV in PAM as described in lines 404 - 414. Furthermore, these limitations for GeCKO screen by using cell-adapted ASFVs in this study were discussed in lines 644 - 650 as suggested.

*4. A previous publication demonstrated the importance of the Niemann-Pick C type 1 (NPC1) endosomal protein in ASFV entry and should be cited and discussed ( PLoS Pathog. 2022 Jan 26;18(1):e1009784. doi: 10.1371/journal.ppat.1009784).*

Thanks for the reviewer’ suggestion. We discussed the study of Cuesta-Geijo MÁ et al. and cite this paper in lines 617 - 620 in the revised manuscript.
